# PREVALENCE OF FIREARM OWNERSHIP AND MENTAL HEALTH CHALLENGES AMONG CAREGIVERS: INSIGHTS FROM A NATIONAL STUDY

**DOI:** 10.1093/geroni/igae098.3135

**Published:** 2024-12-31

**Authors:** Jessika Dayrit, Cesz Islaya, Robyn Medcalf, Lauren Charitou, Renuka Alladi

**Affiliations:** U.S. Department of Veterans Affairs/Palo Alto Healthcare System, Palo Alto, California, United States; U.S. Department of Veterans Affairs/Palo Alto Healthcare System, Palo Alto, California, United States; U.S. Department of Veterans Affairs/Palo Alto Healthcare System, Palo Alto, California, United States; U.S. Department of Veterans Affairs/Palo Alto Healthcare System, Palo Alto, California, United States; U.S. Department of Veterans Affairs/Palo Alto Healthcare System, Palo Alto, California, United States

## Abstract

Despite studies on the association between firearm accessibility and suicide, there is a gap in understanding the prevalence of firearm ownership among caregivers. This study aims to analyze the prevalence of firearm ownership and mental health challenges among civilian and U.S. military veteran caregivers using data from CDC Behavioral Risk Factor Surveillance System (BRFSS) 2021. Employing a cross-sectional study design, with a total sample size of N=17,625, data analysis was conducted using Stata 17, incorporating appropriate weighting and 95% confidence intervals for demographic variables. Binary logistic regression was employed to analyze the data. Findings suggest that both civilian caregivers (OR=1.17, 95% CI=1.07-1.29) and U.S. military veteran caregivers (OR=1.98, 95% CI=1.55-2.54) were more likely to own a firearm at home compared to non-caregivers. Furthermore, civilian caregivers (OR=1.26, 95% CI=1.14-1.40) and U.S. military veteran caregivers (OR=2.14, 95% CI=1.64-2.78) exhibited higher odds of experiencing depression rates, along with an increased likelihood of serious difficulties in concentrating, remembering, or making decisions due to depression (civilian caregivers: OR=1.40, 95% CI=1.23-1.59; U.S. military veteran caregivers: OR=1.65, 95% CI=1.18-2.30). This nationally representative survey highlights critical insights into firearm accessibility, mental health challenges, and safety among caregivers, emphasizing the importance of interventions to prevent unintentional death and suicide. Firearm safety discussions with patients and caregivers help reduce the risk of accidental death or suicidal behavior. Assessment of firearm ownership and safety, as part of the practitioner-patient-caregiver relationship, is as crucial as evaluating chronic illnesses. Promoting comprehensive approaches to addressing firearm safety in clinical practice ensures patient-caregiver well-being.

